# The LIGHT switch: mechanisms of fibroblast pathology in eosinophilic esophagitis

**DOI:** 10.1038/s41385-022-00486-y

**Published:** 2022-02-23

**Authors:** Zoe M. X. Chua, Anne L. Fletcher

**Affiliations:** 1 Department of Biochemistry and Molecular Biology, Monash Biomedicine Discovery Institute, Monash University, Clayton, VIC 3800 Australia; 2 Institute of Immunology and Immunotherapy, University of Birmingham, Edgbaston, B16 2TT UK

New work from Manresa et al. defines LIGHT (TNFSF14)-driven signaling pathways that push fibroblasts to become pathogenic in eosinophilic esophagitis (EoE)^1^. The authors report that fibroblasts switch away from homeostatic WNT signaling towards a dominant pathway of pro-inflammatory non-canonical lymphotoxin beta receptor (LTβR) signaling. These findings in EoE join a strong body of literature across a range of inflammatory diseases, proving that chronically inflamed fibroblasts differentiate into cells that foster immune-mediated pathogenesis and induce harmful tissue contractures.

Fibroblasts are often overlooked as primary drivers of morbidity and mortality in inflammatory disease. Far from an inert scaffold, these are phenotypically malleable cells that respond rapidly to injury and inflammation to mediate tissue healing and a return to homeostasis. When chronically activated in many disease states, their role moves from homeostatic to pathologic, with far-reaching consequences for patients^2^.

A recent study by Manresa et al.^1^ examines the differentiation state of esophageal fibroblasts from patients with active or inactive EoE compared with the esophageal mucosa of healthy donors. EoE is an allergic disease featuring esophageal eosinophil infiltration and a loss of barrier integrity in the esophageal epithelium. Many patients do not respond to pharmacological treatment or prescribed diets, and experience pain, esophageal remodeling, narrowing, and dysphagia as the disease progresses^1,3,4^. The molecular processes that take fibroblasts from homeostatic to pro-inflammatory are of immediate clinical interest in EoE, as well as a range of other inflammatory diseases^2^.

**Fig. 1:**
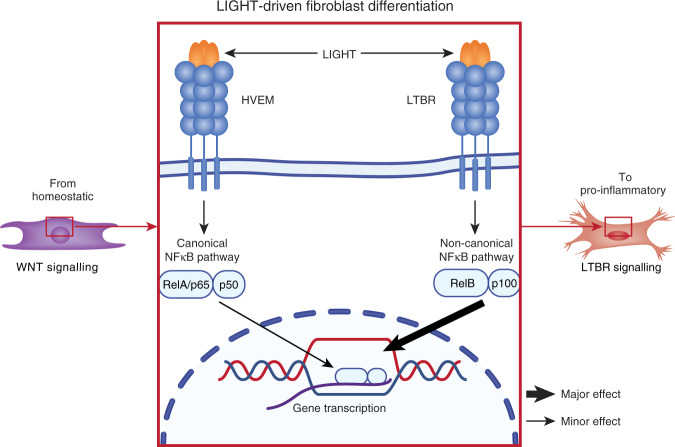
LIGHT-driven fibroblast differentiation. LIGHT imposes a pro-inflammatory phenotype on esophageal fibroblasts by signaling through HVEM or LTβR. LIGHT-HVEM signaling is proposed to activate the canonical NFkB pathway. In the canonical signaling pathway, adaptor proteins are recruited to form the IKK complex, which degrades the IκB inhibitor, leading to nuclear translocation of NF-kB dimers and gene transcription. In contrast, LIGHT-LTβR signaling is thought to mainly occur via the non-canonical NF-kB pathway. In the non-canonical pathway, ligand binding drives NIK to activate IKKα, which phosphorylates the p100 molecule that is then cleaved to produce p52, which translocates into the nucleus as a heterodimer with RelB, where it promotes pro-inflammatory and anti-homeostatic gene expression. Together, these pathways play a primary role in driving homeostatic fibroblasts towards pathogenic pro-inflammatory fibroblasts in eosinophilic esophagitis.

This group previously showed that fibroblast expression of the proinflammatory ligand LIGHT (TNFSF14), was a feature of EoE, and that addition of LIGHT to fibroblast cultures drove upregulation of pro-inflammatory factors^5^. New work now confirms and extends these findings, showing that genes downregulated by LIGHT in culture likely also limit the ability of EoE fibroblasts to maintain esophageal epithelial homeostasis, by downregulating WNT factors (WNT5A, WNT2B), receptors (FZD4), and targets (OLFM2), bone morphogenetic proteins and antagonists (BMP6 and GREM2), semaphorins (SEMA3B and SEMA3D) and insulin growth factor binding proteins (IGFBP3, IGFBP5). The hypothesis therefore is that LIGHT drives fibroblasts to become less able to maintain esophageal homeostasis, and more pro-inflammatory^1^ (Fig. 1).

LIGHT has 3 binding partners: HVEM (herpesvirus entry mediator; TNFRSF14), LTβR and DcR3 (decoy receptor 3; TNFRSF6B)^6^. SiRNA knockdown experiments in esophageal fibroblasts showed that both HVEM and LTβR made distinct contributions to LIGHT-modulated gene expression. LTβR had a dominant role in the transcriptional changes observed. LTβR knockdown abrogated expression of LIGHT-regulated transcripts, including ICAM-1, IL-32, CXCL5, IL-34, and BIRC3, while HVEM knockdown inhibited upregulation of ICAM-1, IL-32, and CXCL5. Consistent with this, basal expression of homeostatic factors (i.e., WNT5A, WNT2B, SEMA3B, and BMP6) was partially restored by loss of either receptor, while upregulation of the WNT inhibitor DKK1 was simultaneously blocked. DcR3 is a decoy receptor that can, among other immunological functions, neutralize LIGHT^6,7^. Its role in this system, if any, was not explored in this paper but would be interesting to test. Together, this data defined the pro-inflammatory and anti-homeostatic phenotype that LIGHT signaling imposed on esophageal fibroblasts.

To further define HVEM’s role in LIGHT-modulated gene expression, the authors overexpressed it (HVEM-OE). In accordance with LIGHT’s pro-inflammatory role, HVEM-OE greatly enhanced ICAM-1 and IL-32 transcripts, while DKK1 in this experiment was upregulated in a LIGHT-independent fashion. The effects of HVEM-OE were largely blocked by LTβR knockdown, which suggests a minor role for HVEM, subordinate to the main driver LTβR. Importantly, the authors then linked HVEM and LTβR signaling to fibroblast-eosinophil tethering in co-culture, creating a direct link between LIGHT signaling and pathology. To test whether LIGHT-mediated upregulation of ICAM-1 enhanced eosinophil-tethering in vitro, eosinophils extracted from whole blood of human donors were co-cultured with LIGHT-treated fibroblasts lacking HVEM or LTβR. siRNA knockdown of either receptor reduced eosinophil-tethering to LIGHT-treated fibroblasts, blocking the formation of multi-eosinophil clusters and highlighting the functional contribution of fibroblasts to eosinophilic pathology in EoE. Imaging of esophageal biopsies using in situ hybridization confirmed that vimentin-positive fibroblasts in EoE upregulate ICAM-1 and downregulate WNT2B^1^. This validated key in vitro observations and was in accordance with the hypothesis that LIGHT drives homeostatic fibroblasts to undergo a pathogenic transformation during EoE, after which they are less able to help maintain epithelial barrier function.

The authors next directly compared the LIGHT-treated in vitro fibroblast transcriptome to the global EoE transcriptome from esophageal biopsies. Co-analysis and comparison of the two transcriptomic databases uncovered 92 differentially expressed genes (DEGs) that both datasets had in common, including upregulation of key hits IL-32, ICAM-1, BIRC3, CXCL5, SAA1, and NIK (NF-κB-inducing kinase, MAP3K14)^1^. Upregulation of these factors is likely to further amplify the pro-inflammatory effects of EoE fibroblasts. For instance, IL-32 is a pro-inflammatory cytokine that stimulates the release of TNF, IL-6, IL-1, and CXCL2^8^. NIK transduces signals as part of the non-canonical pathway downstream of LTβR, suggesting this as a major signaling pathway. A second transcriptomic analysis, comparing in vitro cultured EoE vs healthy fibroblasts, and the changes to normal fibroblasts treated with LIGHT in vitro, yielded similar outcomes. Upregulated targets were again noted pro-inflammatory and LIGHT-driven genes, as well as WNT inhibitors (DKK2), while the common down-regulated targets again included homeostatic factors such as WNT2B and BMP6^1^. These common observations across multiple screens suggest strong concordance between the in vitro transcriptional response of fibroblasts to LIGHT treatment, with the in vivo transcriptome of EoE esophageal biopsies.

To better understand how LIGHT exerted its effects, transcriptomic data from esophageal fibroblasts was fed into a protein interaction database, which predicted contributions from both canonical and non-canonical NF-κB signaling pathways. Accordingly, by Western blot, signal was detected for both nuclear accumulation of p65/RelA, and p100 cleavage into p52, which are hallmarks of canonical and non-canonical signaling respectively. Knockdown of either HVEM or LTβR reduced p65 nuclear accumulation, suggesting both receptors could induce canonical NF-kB signaling, while only LTβR knockdown suppressed non-canonical p100 cleavage^1^.

Lastly, pharmacological inhibitors were used to validate key findings from this paper. BAY11-7082, a canonical NF-κB signaling inhibitor, repressed LIGHT mediated nuclear translocation of p65, whilst the inhibitor of the non-canonical NF-κB pathway, NIK-SMI1, prevented p100 cleavage. Although BAY11-7082 only mildly repressed LIGHT-mediated upregulation of ICAM-1, CD74, CXCL5, and BIRC3, NIK-SMI1 suppressed upregulation of ICAM-1, CD74, and VCAM-1, prevented LIGHT-induced expression of IL-32, IL-34, CXCL5, and BIRC3, and blocked LIGHT-driven suppression of BNP6, GREM2, and SEMA3B. These data show that it may be possible to prevent healthy fibroblasts from progressing towards pathogenic phenotypes through blockade of non-canonical NF-κB2 signaling. It will be of immediate clinical interest to determine whether it is equally possible to reverse the pathogenic fibroblast phenotype once acquired.

T cells are the presumptive source of LIGHT driving fibroblast differentiation in EoE^5^. Mechanistic confirmation of this will be important to pursue to maximize potential therapeutic strategies. Although LIGHT is expressed by a range of infiltrating T cells in EoE, including Th2 cells implicated in this disease, it can also be expressed by myeloid cells, DCs, and NK cells, and there is some evidence it is expressed by eosinophils in mice and humans^9^ (immgen.org). LIGHT also induces the release of inflammatory cytokines from eosinophils^10^, and it fosters the differentiation and survival of pro-allergy Th2 cells^11^.

Collectively, this work shows that LIGHT primarily signals through LTβR to activate the non-canonical NF-κB pathway, which in turn imposes a pro-inflammatory, anti-homeostatic phenotype on esophageal fibroblasts (Fig. 1). There is a supporting role for the canonical NF-κB pathway activated by both HVEM and LTβR. This work adds to a growing body of literature showing that the factors that drive fibroblast functional changes may serve as a platform for new therapeutics. Chronically activated fibroblasts are pathogenic across a startlingly broad range of inflammatory diseases, through autoimmunity, chronic inflammatory disease (e.g., Crohn’s disease, kidney disease), ischemic injury, chronic viral infection, and cancer. Under these conditions, fibroblast subsets emerge bearing distinct, targetable transcriptional profiles^12^. This creates new opportunities to understand the biology of fibroblast development and for therapeutic targeting, with the potential for efficacy across disease indications that generate an enormous burden of disease.
